# Feasibly of CD24/CD11b as a Screening Test for Hematological Malignancies

**DOI:** 10.3390/jpm11080724

**Published:** 2021-07-27

**Authors:** Shiran Shapira, Dina Kazanov, Fatin Mdah, Hadas Yaakobi, Yair Herishanu, Chava Perry, Irit Avivi, Gilad Itchaki, Adi Shacham-Abulafia, Pia Raanani, Mori Hay-Levy, Gal Aiger, Jacob Mashiah, Shahar Lev-Ari, Nadir Arber

**Affiliations:** 1 Integrated Cancer Prevention Center, Tel Aviv Medical Center, Tel Aviv 6423906, Israel; shiransha@tlvmc.gov.il (S.S.); dianak@tlvmc.gov.il (D.K.); mdahfatin@gmail.com (F.M.); hadasyaa@tlvmc.gov.il (H.Y.); morih@tlvmc.gov.il (M.H.-L.); galai@tlvmc.gov.il (G.A.); leva@tauex.tau.ac.il (S.L.-A.); 2 Sackler Faculty of Medicine, Tel Aviv University, Tel-Aviv 6423906, Israel; yairh@tlvmc.gov.il (Y.H.); chavap@tlvmc.gov.il (C.P.); iritavi@tlvmc.gov.il (I.A.); gilad14@gmail.com (G.I.); shacham.adi@gmail.com (A.S.-A.); Piar@clalit.org.il (P.R.); 3 Tel Aviv Medical Center, Department of Hematology, Tel Aviv 6423906, Israel; 4 Davidoff Cancer Center, Rabin Medical Center, Institute of Hematology, Petah Tikva 49100, Israel; 5 Tel Aviv Medical Center, The Pediatric Dermatology Unit, Tel Aviv 6423906, Israel; jacobm@tlvmc.gov.il

**Keywords:** CD24, hematologic cancer, prevention, screening test, sensitivity, specificity, biomarker

## Abstract

An estimated 1.24 million blood cancer cases occur annually worldwide, accounting for approximately 6% of all cancer cases. Currently, there are no standardized hematology cancer screening tests that are recommended for the general population. CD24 is a mucin-like cell surface molecule and P-selectin ligand, which plays a significant role in the maturation of B-lymphocytes and was found to be overexpressed in a number of hematological malignancies. Our primary aim was to assess the sensitivity and specificity of the CD24/CD11b-based blood test for the detection of hematological malignancies. Our cohort included 488 subjects with positive hematological cancer diagnosis (*n* = 122) and healthy subjects (*n* = 366). CD24/CD11b expression in peripheral blood leukocytes (PBLs) obtained from blood samples of participants was analyzed by flow cytometry. Our results demonstrated that the average levels of CD24/CD11b in healthy patients (21.7 ± 9.0) were statistically significantly lower compared to levels of CD24/CD11b in cancer patients (29.5 ± 18.7, *p* < 0.001). The highest levels of CD24/CD11b were found in multiple myeloma (39.1 ± 23.6), followed by chronic myeloid leukemia (33.0 ± 13.7) and non-Hodgkin lymphoma (32.3 ± 13.3). The test had an overall sensitivity for hematologic cancers of 78.5% (95% CI, 70.7–86.3%) and specificity of 80.2% (95% CI, 76.1–84.3%). In conclusion, our findings indicate the feasibility of a CD24/CD11b-based blood test as a screening test of hematological malignancies.

## 1. Introduction

An estimated 1.24 million blood cancer cases occur annually worldwide. More than 700,000 people die from blood cancers every year, accounting for more than 7% of cancer deaths [1]. In the last decade, the understanding of blood cancers pathogenesis has advanced significantly, and as a result, cancer therapy has improved dramatically. Despite these marked improvements, one of the most important prognostic factors for survival is disease staging [2]. Survival increases dramatically if the disease is detected at an early stage when the patient is often still asymptomatic. In solid tumors, the purpose of early detection is to identify the presence of cancer at the early stage, before sending metastases, when the treatment is significantly more likely to be successful [3]. In hematologic cancers, while the benefit of early detection is less pronounced than solid tumors, early detection may significantly enhance treatment efficacy and cure rate. Studies show that when leukemia is detected at an early stage (white blood count less than 50,000 leukocytes, no adverse prognostic signs), a recovery rate of up to 80% can be achieved. In patients diagnosed in an advanced stage (blood count higher than 50,000 leukocytes), recovery rates are reduced to 50% [4]. Similarly, the stage of detection of lymphoma’s, and whether or not the lymphoma has spread outside the lymph system, is an important prognostic factor for survival rates [5].

With the advance of new technologies and the application of efficient and feasible detection platforms, many new biomarkers have been developed and used for diagnosis, classification, and prognostication of hematological malignancies [6]. However, no screening test has been proven to be reliable enough to detect the hematological cancers in their earliest stages prior to the development of symptoms, and there are no standardized hematology cancer screening methods that are recommended for regularly testing the general population [6,7].

CD24 is a highly glycosylated mucin-like GPI-anchored protein. In recent years, it has emerged as an oncogene and a promoter of metastasis. High expression levels of CD24 have been identified in various types of cancers, including hematological malignancies, and indicate a poor prognosis and a more aggressive course of the disease [8]. Our data demonstrate that anti-CD24-based cancer immunotherapy has potential clinical application and might be applicable to hematological malignancies. This was already proven more than two decades ago and supported by other studies showing that anti-B-cell treatment using anti- CD24 and anti-CD21 mAbs was partially efficient in the treatment of bone marrow and organ transplant recipients with B-lymphoproliferative disorders (BLPD) [9]. In pre-clinical studies on acute lymphoblastic leukemia cells, ~88% of leukemic blasts were CD24-positive [10]. A murine ALB9 (IgG1) antibody against CD24 in BLPD achieved complete remission in 61% of the cases, supporting its efficacy. Only limited and transient adverse reactions were observed. We have previously reported that evaluating CD24 levels in peripheral blood leukocytes (PBLs) can serve as a potential promising screening tool to select which healthy subjects are in fact at risk of having CR neoplasia and need to undergo screening colonoscopy [11].

This study summarizes the screening outcomes of blood samples of healthy asymptomatic subjects (*n* = 366) and hematologic cancer patients (*n* = 122) who were screened and completed a full clinical workup at the Integrated Cancer Prevention Center (ICPC) at Tel Aviv Medical Center and division of Hematology at Rabin Medical Center. Our primary aim was to explore whether CD24/CD11b may serve as a biomarker for the early detection of hematological malignancies. Our specific objectives were to assess the sensitivity and specificity of the CD24/CD11b blood test for the early detection of hematologic cancers.

## 2. Materials and Methods

### 2.1. Subjects

Four hundred and eighty-eight blood samples were obtained from healthy volunteers and patients with hematological malignancies, at the age of 20–85 at the hematology, Health Promotion and Integrated Cancer Prevention Center in Tel Aviv Medical Center and division of Hematology at Rabin Medical Center. For each patient with hematological malignancy, three gender and age matched controls were randomly chosen from the data base of the health Promotion Center. Eligible subjects completed a detailed questionnaire on medical history, including personal and family history of cancer, origin, demographic data and other epidemiologic information. Blood specimens were drawn using a standard operating procedure, ensuring uniform handling and collection. Patient information was de-identified and only anonymized data were available to the investigators. Written informed consent was obtained from all eligible participants prior to entry into the study. Approval for this study was provided by the Institutional Review Board (IRB, number 02-130) of Tel Aviv Sourasky Medical Center, Rabin Medical Center and the Israeli Ministry of Health. Subjects were excluded if they did not sign informed consent; or showed symptoms of active inflammation. Neoplastic lesions identified during the workup were classified according to the site and stage in which they were found. The collected data were cross-referenced with the Israeli Cancer Registry, which records all occurrences of neoplasia as is mandated by Israeli law.

### 2.2. Isolation of Peripheral Blood Leukocytes

Blood was collected into standard 9-mL collection tubes (Vacuette^®^, Greiner bio-one, cat. No455036). All samples were collected and processed in an identical manner. Peripheral blood leukocytes were isolated from whole blood samples by collecting buffy coats obtained after blood centrifugation for 3 min at 3000 rpm and discarding the plasma supernatant. Residual erythrocytes were lysed by brief incubation in erythrocyte lysis buffer (ELB) containing 155 mM NH_4_Cl, 0.1 mM EDTA, followed by 30–40 min on ice in 10 mM KHCO_3_. Samples were then centrifuged at 3000 rpm at 4 °C for 5 min. Clean leukocytes pellets were obtained after 1–2 more washes with ELB. PBLs were then fixated with formaldehyde (FA) solution (2% FA in PBS) for 15 min at room temperature (RT) and stored at 4 °C until use.

### 2.3. Flow Cytometry

A total of 1 × 10^6^ leukocytes were used for each test and stained with 0.05 µg anti-CD24-FITC mAb (NS17), anti-CD11b-PerCp-Cy5.5 (rat IgG2b, clone M1/70, Abcam, Israel) or remained unstained for 30 min at RT. NS17 is a humanized IgG1 anti-CD24 antibody that we developed by means of genetic engineering and is fully characterized. The purified NS17 was labeled with FITC using the Pierce™ FITC Antibody Labeling Kit according to manufacturer’s instructions (ThermoFisher Scientific, Waltham, MA, USA).

**Figure 1:**
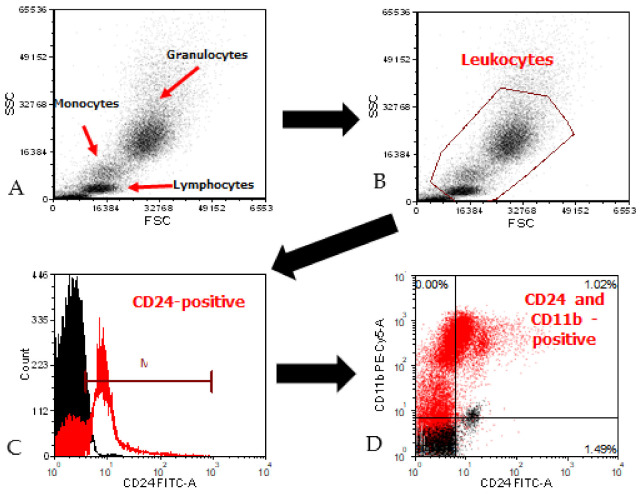
Flow cytometry data analysis. Dot plot of FSC and SSC distinguishes between populations of cells (**A**) and a gate has been applied to identify the leukocytes population (**B**). A total of 30,000 events or cells within the gate were further analyzed and doublet discrimination performed by plotting FSC-H vs. FSC-A. Data are expressed in a histogram to evaluate the relative expression of CD24 (**C**). Two-parameter dot plots display two measurement parameters, FITC (CD24) and PE-Cy5.5 (CD11b) (**D**).

After staining, the cells were washed twice with FACS buffer (0.01% sodium azide, 10% fetal bovine serum [FBS] in ice-cold PBS) and then analyzed by flow cytometry (CyFlow Cube 6, Sysmex, Germany). Data were analyzed following the creation of a hierarchical population tree in the software at the beginning of the screen. This template was used in all subsequent analyses. The template file included compensation adjustment, which was uniformly applied to all the collected data to minimize fluorescence overlap between detection channels. The percentage of positive cells was determined by subtracting the percentage of CD24 and CD11b-positive cells (dual stain) from CD24-positive cells (single stain). Flow cytometry representative graph of the different populations and their fluorescence intensity is detailed in Figure 1.

### 2.4. Statistics

Descriptive statistics (N, mean, standard deviation, proportion) were calculated for all variables, and these are presented in tables by cancer/healthy status as well as graphically via box plots. Logistic regression was performed to assess CD24 count and other variables (gender, age) as predictors of cancer.

ROC analysis was performed to test whether the CD24 levels can discriminate between positive and negative subjects, adjusted to age. An ROC curve and the area under the ROC curve with 95% confidence interval are presented. The area under the ROC curve is a measure of diagnostic accuracy. Sensitivity, specificity, positive predicted value (PPV), and negative predicted value (NPV) were analyzed. The statistical software R (4.0.2) and SPSS 27 (IBM, New York, NY, USA) were used for all the data processing within this study.

## 3. Results

### 3.1. Demographics and Population Characteristics

Demographics and population characteristics of a cohort of 366 healthy subjects and 122 patients with a positive hematological cancer diagnosis, are presented in Table 1. One hundred and eighty-nine (38.7%) subjects were female, 39.3% of the cancer patients and 38.5% in the healthy subjects. The mean age was 65.7 (SD = 15.1) and 54.6 (SD = 10.9) in the cancer and healthy subjects, respectively. Of the cancer patients, 32.0% had not been treated or undergone surgical procedure (virgin cancer).

**Figure 2:**
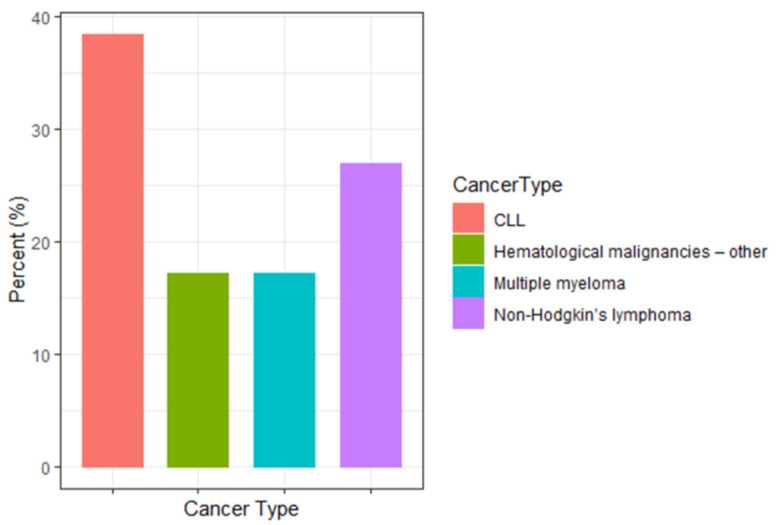
Cancer Type distribution. Cancer types were categorized into major cancer groups. The main groups included CLL (37.9%), non-Hodgkin’s lymphoma (27.4%), and multiple myeloma (16.9%). Hematological malignancies-other (17.2%) included: myelofibrosis (1.6%), acute lymphocytic leukemia (3.3%), acute myeloid leukemia (4.9%), chronic myeloid leukemia (0.8%), and Hodgkin’s lymphoma (4.1%) and unspecified lymphoma (2.5%).

Cancer types were categorized into major cancer groups (Figure 2). The main groups included chronic myeloid leukemia (CLL) (38.5%), non-Hodgkin lymphoma (27.0%), and multiple myeloma (17.2%).

### 3.2. CD24 Levels in Healthy and Cancer Subjects

CD24/CD11b expression in 488 eligible participants was analyzed, and 122 of them were patients with a positive hematological cancer diagnosis. Flow cytometry analysis showed levels of CD24/CD11b expression in PBLs obtained from study participants.

**Figure 3:**
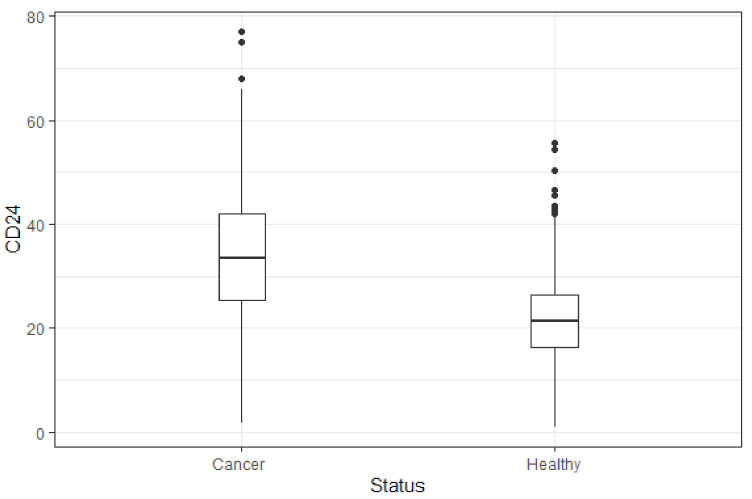
Box plot of the CD24 levels in healthy subjects versus cancer patients. Flow cytometry analysis showed levels of CD24/CD11b expression in PBLs obtained from study participants.

Figure 3 presents a box plot of the CD24/CD11b levels in healthy subjects versus cancer patients. Flow cytometry analysis showed levels of CD24/CD11b expression in PBLs obtained from study participants. The average levels of CD24/CD11b in healthy patients (21.7 ± 9.0) were statistically significantly lower compared to in cancer patients (29.5 ± 18.7, *p* < 0.001). Sensitivity analysis found that the test score was not affected by age (*p* = 0.560).

### 3.3. CD24 Levels in Blood Cancer Subjects

**Figure 4:**
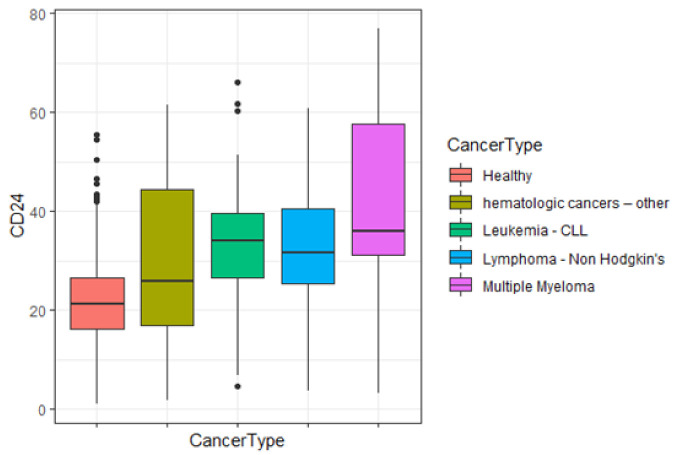
Box plot of CD24 levels by major cancer groups. Hematological cancer types were categorized into major groups: CLL, non-Hodgkin’s lymphoma, multiple myeloma and hematological malignancies-other. Flow cytometry analysis showed levels of CD24/CD11b expression in PBLs obtained from study participants.

CD24/CD11b expression in 122 cancer patients having different types of hematological cancers was analyzed. Figure 4 presents a box plot of the CD24/CD11b levels in the 3 major cancer types. The highest levels of CD24 were found in multiple myeloma (39.1 ± 23.6), followed by CLL (33.0 ± 13.7) and non-Hodgkin lymphoma (32.3 ± 13.3). No significant differences were found between CD24/CD11b levels of healthy subjects compared to patients with other hematological cancers. Interestingly, in this group, all the patients were treated (non-virgin cancer).

### 3.4. Sensitivity and Specificity of CD24 Screening Test

**Figure 5:**
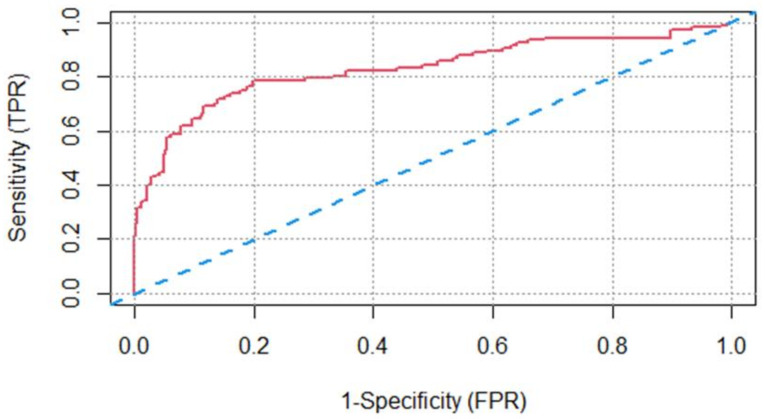
Receiver operating characteristic curve (ROC) of CD24 blood test. The top curve uses a CD24 score cutoff (AUC = 0.83). The bottom curve is what would be expected by chance alone (AUC = 0.50).

In determining the specificity and sensitivity of the CD24/CD11b test and its ability to discriminate between patients with cancer from healthy individuals, area under the receiver operator curve (ROC), cutoff value was derived. Figure 5 presents the ROC curve of the CD24/CD11b on the study population. The AUC of the CD24/CD11b screening test was 0.830; (95% CI, 0.778–0.881).

Table 2 includes the sensitivity, specificity, positive predicted value (PPV), and negative predicted value (NPV) for the study cohort. The test had an overall sensitivity for hematologic cancer of 78.5% (95% CI, 70.7% to 86.3%) and specificity of 80.2% (95% CI, 76.1% to 84.3%).

Higher specificity values were obtained using higher CD24/CD11b cutoff values: specificity of 90.1% and sensitivity 53.3% (cutoff CD24/CD11b = 32.7); and specificity of 95% and sensitivity of 33.6% (cutoff CD24/CD11b = 37.7).

## 4. Discussion

A significant difference between the expression of CD24/CD11b levels in the PBLs of hematological cancer patients and healthy subjects is presented. Especially, a high level of the CD24/CD11b score was found in subjects with CLL, non-Hodgkin’s lymphoma and multiple myeloma. Sensitivity and specificity values of the CD24 blood test indicated feasibility of CD24/CD11b to serve as a cancer screening test for further development of hematological neoplasia.

Screening tests are used to determine whether an asymptomatic individual has cancer. Screening for colon, breast, cervical and prostate cancers are good examples of the potential of effective screening methods to significantly reduce morbidity and mortality [12]. To date, there is no screening test for hematological malignancies. Several molecular testing and biomarkers have been evaluated for detection and diagnosis of hematologic cancers. These include: somatic mutations in preleukaemic hematopoietic stem and progenitor cells (HSPCs), presence of tumor-specific mutations in cell-free DNA (cfDNA) and circulating tumor DNA (ctDNA) [7,13,14,15]. In our study, an increase in the peripheral blood levels of CD24/CD11b was found among subjects with hematologic malignancies compared to healthy subjects (Figure 3). Our sensitive analysis demonstrated that this statistically significant difference was not affected by age or gender, and therefore indicate the rationale for assessing CD24 as a possible biomarker for hematological malignancies.

Several studies have shown that increased expression of CD24 is associated with tumor progression [16,17,18,19,20]. Elevated levels of CD24 were found to be associated with the spread of the tumor and the formation of metastases [20,21,22,23]. In our study, we have found elevated levels of CD24 in multiple myeloma, CLL and non-Hodgkin lymphoma compared to CD24 levels of healthy subjects (Figure 4). Our results are consistent with previous studies demonstrating the role of CD24 in recurrent follicular lymphoma [24], in R-CHOP treatment response and tumor immunosuppression in diffuse large B-cell lymphoma [25] and in multiple myeloma tumorigenicity [26]. These findings indicate the utility of CD24 expression in a wide variety of hematological cancers.

In many diagnostic tests, there is a trade-off between specificity and sensitivity. Most regulatory authorities demand that the test must have very high specificity; otherwise, too many healthy individuals will receive a positive test that may lead to unnecessary (invasive) follow-up procedures and cause anxiety [3,27]. The CD24/Cd11b test had an overall sensitivity of 78.5% (95% CI, 70.7–86.3%) and specificity of 80.2% (95% CI, 76.1–84.3%). Higher specificity values of 90% and 95% (sensitivity values of 53.3% and 33.6%, accordingly) were obtained with different CD24/Cd11b cutoff values. These data are preliminary and require further development and optimization.

Our study has several limitations. First, the analysis was performed on a relatively small sample size. Larger cohorts of healthy subjects need to be followed prospectively in order to verify the accuracy of the suggested test. Second, we did not assess other risk factors that may be associated with elevated risk of blood cancers, which could confound or mediate the relationship between CD24 and prevalence of cancer. Third, the test was not able to identify the specific type for hematological cancer. On the other hand, the test has the potency to be part of a surveillance program with additional biomarkers for hematological malignancies. It can also serve as a prognostic and/or predictive marker.

## 5. Conclusions

A significant increase in the levels of CD24/CD11b levels was found among patients with hematological cancers, as compared to healthy subjects. Sensitivity and specificity are encouraging for the feasibility of the CD24/CD11b blood test for further development and validation as a hematologic cancer screening test and as an early marker for these diseases.

## Figures and Tables

**Table 1:** Demographic and population characteristics of the participants (*N* = 488).

Characteristic		Cancer (*N* = 122)	Healthy (*N* = 366)
		*N* (%) or Mean (SD)	*N* (%) or Mean (SD)
Female	Gender	48 (39.3%)	141 (38.5) ^NS^
Male		74 (60.7%)	225 (61.5%)
	Age	65.7 (15.1)	54.6 (10.9) *
No treatment (virgin cancer)	Cancer Treatments	39 (32.0%)	
Had treatment		83 (68%)	
CLL	Cancer Type	47 (38.5)	
Non-Hodgkin’s lymphoma		33 (27.0)	
Multiple myeloma		21 (17.2)	
Hematological malignancies—other		21 (17.2)	

SD—standard deviation; * difference between groups, *p* < 0.001, NS—difference between groups is non-significant. Hematological malignancies—other (17.2%) included: myelofibrosis (1.6%), acute lymphocytic leukemia (3.3%), acute myeloid leukemia (4.9%), chronic myeloid leukemia (0.8%), and Hodgkin’s lymphoma (4.1%) and unspecified lymphoma (2.5%).

**Table 2:** Sensitivity, specificity, positive predicted value (PPV), negative predicted value (NPV) analysis.

	% (*n*/*N*)	95% CI
Accuracy	79.83% (376/471)	[79.8%;79.9%]
Sensitivity	78.5% (84/156)	[70.7%;86.3%]
Specificity	80.2% (292/364)	[76.1%;84.3%]
PPV	53.8% (84/156)	[46.0%;61.7%]
NPV	92.7% (292/315)	[89.8%;95.6%]

Accuracy—overall accuracy, PPV—positive predicted value, NPV–negative predicted value, CI—confidence interval.

## Data Availability

The data presented in this study are available on request from the corresponding author.
